# Association of the major depressive disorder-associated locus rs12415800 with bipolar disorder in a Taiwan Biobank–NHIRD linked cohort

**DOI:** 10.1192/bjo.2026.12114

**Published:** 2026-10-10

**Authors:** Mu-N Liu, Kan-Chih Chen, Ding-Lieh Liao, Mu-Hong Chen, Po-Hsiu Kuo, Shih-Jen Tsai

**Affiliations:** Department of Psychiatry, Taipei Veterans General Hospital, Taipei, Taiwan; Department of Psychiatry, College of Medicine, National Yang Ming Chiao Tung University, Taipei, Taiwan; Department of General Psychiatry, Taoyuan Psychiatric Center, Taoyuan City, Taiwan; Department of Public Health & Institute of Epidemiology and Preventive Medicine, National Taiwan University, Taipei, Taiwan; Psychiatric Research Center, Wan Fang Hospital, Taipei Medical University, Taipei, Taiwan

**Keywords:** *SIRT1*, major depressive disorder, bipolar disorder, Taiwan Biobank, National Health Insurance Research Database

## Abstract

**Background:**

Genome-wide association studies in East Asian populations have identified the gene variant, rs12415800, of the *Sirtuin 1* gene (*SIRT1*) as a major depressive disorder (MDD) risk locus with the A allele conferring increased risk. Accumulating evidence suggests that *SIRT1*-related genetic effects extend beyond MDD.

**Aims:**

To investigate the association between rs12415800 and bipolar disorder, with additional analysis of MDD.

**Method:**

We analysed data from the Taiwan Biobank linked to the National Health Insurance Research Database, including 145 780 participants of Han Chinese ancestry. Clinically diagnosed bipolar disorder and MDD were identified using standardised ICD-9-CM and ICD-10-CM codes. Multivariable logistic regression models were applied with the GG genotype as the reference, adjusting for age and sex. Stratified analyses were conducted by age group and sex.

**Results:**

Both the rs12415800 AG and AA genotypes were associated with an increased risk of bipolar disorder. Compared with GG genotype carriers, AA genotype carriers had a significantly higher risk of bipolar disorder overall (adjusted odds ratio (aOR) = 1.20, 95% CI = 1.05–1.37), particularly among participants aged ≥50 years (aOR = 1.38, 95% CI = 1.14–1.68). The overall and age-stratified associations remained materially similar after excluding participants with schizophrenia or schizo-affective disorder, although sex-specific findings were inconsistent.

**Conclusions:**

Our findings suggest that genetic variation near *SIRT1*, particularly the rs12415800 risk A allele, may be associated with bipolar disorder susceptibility, especially among older individuals. Given prior evidence linking this allele to reduced *SIRT1* mRNA expression, further investigation of SIRT1-related pathways in the pathophysiology of bipolar disorder is warranted.

Mood disorders, including major depressive disorder (MDD) and bipolar disorder, are leading contributors to global disability and are associated with substantial personal, societal and economic burdens.^1,2^ Despite extensive research, the etiopathogenesis of mood disorders remains to be completely understood, with significant clinical heterogeneity and complex interactions among genetic susceptibility and environmental exposures.^3,4^ Twin and family studies have consistently demonstrated a moderate to high heritable component for mood disorders, motivating large-scale genomic efforts to identify susceptibility loci and biological pathways involved in the risk of developing mood disorders.^3,5^

Genome-wide association studies (GWAS) have substantially advanced understanding of the genetic architecture of mood disorders.^6,7^ However, many loci identified in populations of European ancestry show limited transferability to East Asian populations, underscoring the importance of ancestry-specific investigations.^8^ In this context, a landmark GWAS conducted in Han Chinese populations identified a single nucleotide polymorphism (SNP), rs12415800, located near the *Sirtuin 1 (SIRT1)* gene on chromosome 10, as one of the first GWAS significant risk variants for MDD.^9^ Notably, the risk allele (A) frequency of rs12415800 is markedly higher in East Asian populations than in Europeans,^9,10^ suggesting population-specific genetic contributions to mood disorder susceptibility. *SIRT1* encodes a nicotinamide adenine dinucleotide (NAD^+^)-dependent deacetylase that plays a critical role in cellular energy metabolism, circadian rhythm regulation, neurodevelopment, synaptic plasticity and stress responsiveness.^11,12^ Experimental and translational studies have implicated *SIRT1* signalling in neurobiological processes relevant to mood regulation, including neuronal resilience, dendritic complexity and neuroinflammatory responses.^11,13–16^ Recent clinical and molecular evidence supports the role of SIRT1 in depression. *SIRT1* gene variants, including rs12415800, have been associated with depressive symptoms in patients with type 2 diabetes. These associations were accompanied by reduced peripheral *SIRT1* mRNA expression, suggesting that *SIRT1* dysregulation may contribute to depression-related pathophysiology.^17^

In addition to genetic association evidence, converging human neuroimaging studies further support a role for *SIRT1* in mood disorder-related brain alterations. Human neuroimaging and post-mortem studies suggest that the rs12415800 risk allele (A) is associated with altered brain structure and reduced *SIRT1* expression in regions implicated in affective and cognitive processing, providing biological plausibility for its involvement in mood pathology.^18^ A study of first-episode, medication-naive patients with MDD reported that rs12415800 was associated with altered grey matter volume in brain regions implicated in affective and cognitive processing. This finding suggests that *SIRT1*-related genetic variation may influence early neuroanatomical substrates of MDD.^19^ Complementing these findings, diffusion-based network analyses in adolescent patients with MDD have shown that rs12415800 is associated with disruptions in white matter neural networks. These results indicate potential effects on brain connectivity and information integration during neurodevelopmentally sensitive periods.^20^ Together, these neuroimaging observations provide translational evidence linking *SIRT1* genetic variation to both grey matter structure and white matter network organisation, supporting a biologically plausible pathway through which *SIRT1*-related loci – such as rs12415800 – may contribute to vulnerability to mood disorders.

Although prior studies have linked *SIRT1*-related variants to MDD, suicidality, schizophrenia and personality traits associated with depressive symptoms, evidence regarding bipolar disorder remains limited and inconsistent.^13,21–23^ Existing studies have often been constrained by modest sample sizes, case–control designs, lack of age- or sex-stratified analyses and limited capacity to examine clinically diagnosed outcomes in real-world populations. Therefore, large ancestry-specific biobank studies linked to longitudinal health records are needed to clarify whether rs12415800 contributes to bipolar disorder risk in East Asian populations.

The Taiwan Biobank (TWB), when linked with the National Health Insurance Research Database (NHIRD), offers a unique opportunity to address these gaps. TWB provides high-quality genomic data and detailed baseline phenotyping in a large community-based cohort, while NHIRD enables long-term, population-wide follow-up for clinically diagnosed mood disorders using standardised diagnostic coding.^24,25^ This integrated resource allows for robust evaluation of genetic associations with incident mood disorders, subtype-specific outcomes and potential sex- or age-dependent effects in an East Asian population.

In this study, we leverage TWB–NHIRD linked data to investigate the association between MDD GWAS locus rs12415800 and mood disorders, with a focus on clinically diagnosed MDD and bipolar disorder. Despite extensive research, the etiopathogenesis of mood disorders remains to be completely understood, with significant clinical heterogeneity and complex interactions among genetic susceptibility and environmental exposures. By integrating genetic information with longitudinal health records, we aim to clarify the role of this East Asian–enriched genetic variant in mood disorder susceptibility and to contribute population-specific evidence to the genetic epidemiology of mood disorders.

## Method

### Study population

Participants were drawn from the TWB, a nationwide prospective cohort that recruits community-dwelling adults aged ≥20 years across Taiwan. At enrolment, all participants provided written informed consent, completed standardised questionnaires, underwent physical examinations and donated biological specimens for genomic analysis.^26^ Individuals with incomplete genotype data, missing key covariates, or evidence of non-Han Chinese ancestry were excluded. To ascertain longitudinal psychiatric outcomes, TWB participants were linked to the NHIRD using encrypted personal identifiers. The NHIRD is a nationwide claims database derived from Taiwan’s single-payer National Health Insurance programme, which covers nearly the entire Taiwanese population and includes longitudinal records of in-patient and out-patient diagnoses, procedures and prescriptions.^25^ TWB enrolment data were successfully linked to NHIRD claims records, with psychiatric outcomes ascertained during the available NHIRD observation period from 1 January 2000 to 31 December 2021. Ultimately, the analytic sample comprised 145 780 TWB participants enrolled between 10 December 2008 and 31 December 2021 who passed genotyping quality control.

### Genotyping and quality control

Blood samples for genomic DNA extraction were collected from participants at the baseline TWB enrolment visit, during which standardised questionnaires, physical examinations and biological specimen collection were conducted. Genotyping was performed using customised Axiom Genome-Wide Array chips designed for the TWB, followed by standard quality control procedures at both the sample and variant levels. At the sample level, individuals with poor genotyping quality, sex inconsistency, excessive missingness, or close relatedness were excluded. At the variant level, SNPs were filtered according to standard quality control criteria, including SNP call rate, Hardy–Weinberg equilibrium and minor allele frequency thresholds. The SNP rs12415800, located near the *SIRT1* gene on chromosome 10, was successfully genotyped and passed all quality control criteria. Genotypes were coded additively according to the number of rs12415800 risk alleles.

### Ascertainment of mood disorders

Mood disorder outcomes were identified from the NHIRD using ICD diagnostic codes recorded in in-patient and out-patient claims. The standard ICD-9-CM and ICD-10-CM code algorithms were used to identify MDD (296.2, 296.3 (ICD-9-CM); F32, F33 (ICD-10-CM)) and bipolar disorder (296.0, 296.1, 296.4–296.7, 296.80, 296.81, 296.89 (ICD-9-CM); F30, F31 (ICD-10-CM)). Consistent with prior NHIRD research, cases were defined as participants with at least two out-patient diagnoses recorded by psychiatrists or at least one in-patient diagnosis of the relevant disorder.^27,28^ For participants meeting the out-patient criterion, the index date was defined as the date of the first qualifying out-patient diagnosis, where this date represents the earliest clinically documented occurrence of the disorder. For participants meeting the in-patient criterion, the index date was defined as the date of the first qualifying in-patient diagnosis. Participants without any recorded mood disorder diagnosis throughout the follow-up period were treated as controls. For the sensitivity analysis, schizophrenia and schizo-affective disorder were identified using ICD-9-CM code 295.x and ICD-10-CM codes F20 and F25, applying the same requirement of at least two psychiatrist-recorded out-patient diagnoses or one in-patient diagnosis.

### Covariates

Baseline covariates, including education, smoking status and alcohol use, were obtained from standardised TWB questionnaires administered at enrolment. Psychiatric outcomes were not derived from questionnaires but from NHIRD diagnostic claims.

### Statistical analysis

Descriptive statistics were used to summarise baseline characteristics based on the rs12415800 genotype. Differences were assessed using *χ* ^2^ tests for categorical variables and analysis of variance for continuous variables.

The association between rs12415800 and incident mood disorders was examined using multivariable logistic regression models. Odds ratios and 95% confidence intervals were estimated, and all models were adjusted for age and sex. Because *SIRT1*-related pathways are implicated in ageing, cellular stress responses and sex-related biological processes,^20,29,30^ stratified analyses were conducted by sex and by age group. Age 50 years was selected *a priori* as an approximate midlife threshold because midlife is commonly conceptualised as a pivotal life-course period spanning approximately ages 40–60 or 40–65 years, during which ageing-related biological, psychological and functional changes begin to accumulate, while also preserving adequate statistical power in both strata.^31^ Statistical significance was defined using two-sided *P* values. To address potential diagnostic overlap and reduce confounding by other psychotic disorders, a sensitivity analysis excluded all participants meeting the above claims-based definition of schizophrenia or schizo-affective disorder from the study sample, including both mood-disorder cases and controls.

## Results

### Participant characteristics

The study included 145 780 participants genotyped for rs12415800 (47 179 GG; 71 656 AG; 26 945 AA). Baseline characteristics were largely comparable across genotype groups (Table 1). Small but statistically significant differences were observed in sex distribution and mean age; however, no differences were noted in education level, smoking status or alcohol consumption.

**Table 1:** Baseline characteristics of the study participants according to rs12415800 genotype Table 1 long description.

rs12415800	GG (*n* = 47 179)	AG (*n* = 71 656)	AA (*n* = 26 945)	*p*
Male (*n*, %)	17 313 (36.70)	25 787 (35.99)	9637 (35.77)	**0.01**
Age, mean, s.d.	49.52 ± 11.42	49.41 ± 11.35	49.31 ± 11.40	**0.04**
Education (*n*, %)				0.11
No/non-formal	93 (0.20)	160 (0.22)	43 (0.16)	
Elementary school	2254 (4.78)	3266 (4.56)	1268 (4.71)	
Junior high school	3493 (7.40)	4994 (6.97)	1862 (6.91)	
Senior high/vocational school	13 385 (28.37)	20 479 (28.58)	7705 (28.60)	
College/university	22 675 (48.06)	34 648 (48.35)	12 993 (48.22)	
Graduate school	5279 (11.19)	8109 (11.32)	3074 (11.41)	
Smoking (*n*, %)	13 044 (27.65)	19,485 (27.20)	7280 (27.02)	0.12
Drinking (*n*, %)	2863 (6.07)	4360 (6.08)	1635 (6.07)	0.99

Statistical significance is indicated with bold type.

### Association between rs12415800 and MDD

The distribution of rs12415800 adheres to Hardy–Weinberg equilibrium (*P* = 0.363). In the overall sample, rs12415800 was not significantly associated with MDD after adjustment for age and sex (Table 2). Compared with the GG genotype, neither AG (adjusted odds ratio (aOR) = 0.99, 95% CI 0.96–1.03) nor AA (aOR = 1.03, 95% CI 0.98–1.08) showed a significant association with MDD.

**Table 2:** Association between rs12415800 and major depressive disorder (MDD) Table 2 long description.

rs12415800	Non-MDD (*n*, %)	MDD (*n*, %)	Odds ratio (95% CI)	aOR (95% CI)
All				
GG	42 189 (89.42)	4990 (10.58)	Ref.	Ref. (Adjusted sex and age)
AG	64 099 (89.45)	7557 (10.55)	1.00 (0.96–1.04)	0.99 (0.96–1.03)
AA	24 011 (89.11)	2934 (10.89)	1.03 (0.98–1.08)	1.03 (0.98–1.08)
Age ≥50				
GG	21 726 (88.60)	2796 (11.40)	Ref.	Ref. (Adjusted sex)
AG	32 721 (88.39)	4297 (11.61)	1.02 (0.97–1.07)	1.01 (0.97–1.07)
AA	12 179 (87.83)	1688 (12.17)	**1.08 (1.01–1.15)**	**1.07 (1.01–1.14)**
Age <50				
GG	20 463 (90.32)	2194 (9.68)	Ref.	Ref. (Adjusted sex)
AG	31 378 (90.59)	3260 (9.41)	0.97 (0.92–1.03)	0.97 (0.91–1.02)
AA	11 832 (90.47)	1246 (9.53)	0.98 (0.91–1.06)	0.98 (0.91–1.05)
Male				
GG	15 974 (92.27)	1339 (7.73)	Ref.	Ref. (Adjusted age)
AG	23 728 (92.02)	2059 (7.98)	1.04 (0.96–1.11)	1.04 (0.96–1.11)
AA	8912 (92.48)	725 (7.52)	0.97 (0.88–1.07)	0.97 (0.88–1.07)
Female				
GG	26 215 (87.78)	3651 (12.22)	Ref.	Ref. (Adjusted age)
AG	40 371 (88.01)	5498 (11.99)	0.98 (0.94–1.02)	0.98 (0.94–1.02)
AA	15 099 (87.24)	2209 (12.76)	1.05 (0.99–1.11)	**1.05 (1.00–1.11)**

Ref., reference group; aOR, adjusted odds ratio.

Statistical significance is indicated with bold type.

Age-stratified analyses revealed a modest association in participants aged ≥50 years, in whom the AA genotype was associated with a slightly increased risk of MDD (aOR = 1.07, 95% CI 1.01–1.14), whereas no significant associations were observed in those aged <50 years. Sex-stratified analyses showed no significant associations in either males or females.

### Association between rs12415800 and bipolar disorder

As shown in Table 3, the *SIRT1* rs12415800 polymorphism was significantly associated with bipolar disorder in the overall sample. Using the GG genotype as the reference, individuals carrying the AG genotype had a modest but statistically significant increase in bipolar disorder risk (adjusted odds ratio (aOR) = 1.12, 95% CI = 1.01–1.25), while those with the AA genotype showed a stronger association (aOR = 1.20, 95% CI = 1.05–1.37) after adjustment for age and sex.

**Table 3:** Association between rs12415800 and bipolar disorder Table 3 long description.

rs12415800	Non-bipolar disorder (*n*, %)	Bipolar disorder (*n*, %)	Odds ratio (95% CI)	aOR (95% CI)
All				
GG	46 662 (98.90)	517 (1.10)	Ref.	Ref. (adjusted sex and age)
AG	70 775 (98.77)	881 (1.23)	**1.12 (1.01–1.25)**	**1.12 (1.01–1.25)**
AA	26 591 (98.69)	354 (1.31)	**1.20 (1.05–1.38)**	**1.20 (1.05–1.37)**
Age ≥50				
GG	24 286 (99.04)	236 (0.96)	Ref.	Ref. (adjusted sex)
AG	36 582 (98.82)	436 (1.18)	**1.23 (1.05–1.44)**	**1.22 (1.04–1.44)**
AA	13 683 (98.67)	184 (1.33)	**1.38 (1.14–1.68)**	**1.38 (1.14–1.68)**
Age <50				
GG	22 376 (98.76)	281 (1.24)	Ref.	Ref. (adjusted Sex)
AG	34 193 (98.72)	445 (1.28)	1.04 (0.89–1.20)	1.04 (0.89–1.20)
AA	12 908 (98.70)	170 (1.30)	1.05 (0.87–1.27)	1.05 (0.86–1.27)
Male				Adjusted age
GG	17 148 (99.05)	165 (0.95)	Ref.	Ref.
AG	25 507 (98.91)	280 (1.09)	1.14 (0.94–1.38)	1.14 (0.94–1.38)
AA	9510 (98.68)	127 (1.32)	**1.34 (1.10–1.75)**	**1.39 (1.10–1.75)**
Female				Adjusted age
GG	29 514 (98.82)	352 (1.18)	Ref.	Ref.
AG	45 268 (98.69)	601 (1.31)	1.11 (0.98–1.27)	1.11 (0.97–1.27)
AA	17 081 (98.69)	227 (1.31)	1.11 (0.94–1.32)	1.11 (0.94–1.32)

Ref., reference group; aOR, adjusted odds ratio.

Statistical significance is indicated with bold type.

Age-stratified analyses revealed that these associations were driven primarily by participants aged ≥50 years. In this older subgroup, both AG (aOR = 1.22, 95% CI = 1.04–1.44) and AA genotypes (aOR = 1.38, 95% CI = 1.14–1.68) were significantly associated with increased bipolar disorder risk. In contrast, no significant associations were observed among participants aged <50 years (Table 3).

Sex-stratified analysis revealed a sex effect. Among males, the AA genotype was significantly associated with bipolar disorder (aOR = 1.39, 95% CI = 1.10–1.75), whereas the AG genotype did not reach statistical significance. Among females, neither the AG nor AA genotype was significantly associated with bipolar disorder after age adjustment (Table 3).

Overall, these findings suggest an age- and sex-dependent association between rs12415800 and bipolar disorder, with the strongest effects observed in older individuals and in males carrying the AA genotype.

### Mean age at first recorded NHIRD diagnosis of MDD or bipolar disorder

Mean age at first recorded NHIRD diagnosis of MDD and bipolar disorder did not differ significantly across rs12415800 genotypes in the overall sample (Supplementary Table S1).

Overall, rs12415800 was selectively associated with bipolar disorder, with evidence suggesting that the GG genotype confers a lower risk of bipolar disorder, particularly among older individuals and males. No consistent associations were observed with age at first recorded NHIRD diagnosis.

Furthermore, to address possible diagnostic overlap and reduce potential confounding by psychotic disorders, we conducted a sensitivity analysis excluding all participants with schizophrenia or schizo-affective disorder diagnoses from the study sample. The overall and age-stratified associations between rs12415800 and bipolar disorder remained materially similar: the AG and AA genotypes remained significantly associated with bipolar disorder overall and among participants aged ≥50 years, whereas neither genotype was significant among those aged <50 years. However, the sex-stratified pattern differed from the primary analysis. The association between the AA genotype and bipolar disorder in males was no longer statistically significant after exclusion (aOR = 1.29, 95% CI = 0.99–1.67), while the AG genotype was significantly associated with bipolar disorder in females (aOR = 1.18, 95% CI = 1.02–1.36); the AA genotype remained non-significant in females (Supplementary Tables S2–S4).

## Discussion

In this large, population-based study leveraging linkage between the TWB and the NHIRD, we investigated the association between the rs12415800 variant near *SIRT1* and clinically diagnosed mood disorders in a Han Chinese population. We found both AG and AA genotypes were linked to an elevated risk of bipolar disorder. Individuals with the AA genotype had a markedly elevated risk relative to those with the GG genotype, suggesting a protective effect associated with the GG genotype (Table 3). The protective association of the GG genotype was more significant in males and subjects aged 50 and above. Conversely, rs12415800 exhibited no significant association with MDD in the total sample, with the exception of a minor association observed in the elderly participants (Table 2). By integrating rs12415800 genotyping with longitudinal, claims-based psychiatric outcomes, our findings provide robust population-level evidence supporting rs12415800 as a susceptibility locus for mood disorders, especially bipolar disorder, in Han Chinese populations. Our results extend a prior GWAS study that identified rs12415800 as a GWAS significant locus for MDD in Han Chinese women (odds ratio: 1.167), highlighting the value of reducing phenotypic heterogeneity to uncover genetic signals.^9^

In contrast to the initial GWAS findings in Han Chinese populations,^32^ we did not observe a significant association between rs12415800 and MDD in the overall sample, with the exception of a modest effect detected among older individuals (Table 2). This discrepancy is not unexpected and likely reflects differences in case ascertainment and phenotypic composition between population-based cohorts and earlier GWAS study. The original association between rs12415800 and MDD was identified by the CONVERGE consortium, which focused on women with recurrent, hospital-treated and predominantly melancholic depression – a clinically severe and genetically enriched subtype.^32^ In contrast, MDD subjects identified through claims-based data in the present study likely encompass a broader spectrum of severity, including milder or environmentally driven forms of MDD, which may dilute detectable genetic effects. Notably, the weak association observed in participants aged ≥50 years could be due to age-related vulnerability in biological pathways regulated by *SIRT1*, such as mitochondrial function, circadian regulation,and stress resilience.^11,12^ Together, these findings suggest that the influence of rs12415800 on MDD risk may be context-dependent, emerging primarily in clinically severe or late-life MDD rather than across heterogeneous, population-ascertained depressive phenotypes.

The biological plausibility of rs12415800 involvement in mood disorders is supported by converging evidence from molecular, neuroimaging and animal studies. *SIRT1* encodes a NAD^+^-dependent deacetylase that plays a central role in cellular energy homeostasis, circadian rhythm regulation, neurodevelopment and stress-related signalling pathways.^11,12,33^ These processes are increasingly recognised as core components of mood disorder pathophysiology. Experimental studies have demonstrated that *SIRT1* influences synaptic plasticity, dendritic complexity and neuronal resilience, while human studies have reported associations between the rs12415800 risk allele (A) and reduced grey matter volume in regions implicated in affective regulation, including the cerebellum, prefrontal cortex and precuneus.^14,18,19^ Moreover, reduced *SIRT1* mRNA expression associated with the risk allele (A) in post-mortem brain tissue and peripheral blood provides functional support linking this genetic variant to downstream biological effects.^18^

Our findings also underscore the importance of population-specific genetic architecture in psychiatric disorders. The rs12415800 risk allele (A) is common in East Asian populations but rare in individuals of European ancestry, which likely contributes to inconsistent replication across ancestries in large international GWAS meta-analyses.^9,10^ This observation aligns with a growing body of evidence indicating that a substantial proportion of genetic liability for mood disorders is ancestry-dependent, particularly for common variants with modest effect sizes.^3^ The present study reinforces the value of large-scale biobanks in East Asian populations for identifying and validating genetic risk loci that may be under-represented or absent in European-dominated data-sets.

The association between rs12415800 and bipolar disorder was more significant in those aged 50 years or older, while no notable genetic effect was detected in those under 50 years (Table 3). The stronger association in older adults may reflect age-related biological contexts in which *SIRT1*-related pathways – such as energy metabolism, circadian regulation and stress responsiveness – become increasingly relevant as neuronal resilience declines with ageing.^30,34^ In contrast, bipolar disorder with earlier onset may be more strongly driven by neurodevelopmental or environmental factors, thereby attenuating the detectable impact of common genetic variants with modest effect sizes.^35^ Our findings support a context-dependent role of rs12415800 in bipolar disorder risk, modified by age, and highlight the importance of stratified analyses in genetic studies of mood disorders.

An earlier case–control study conducted in a Japanese cohort examined four tagging variants within the *SIRT1* gene and identified an association between *SIRT1* gene variants and schizophrenia but found no significant association with bipolar disorder.^21^ The divergent findings between the Japanese study, which found an association between *SIRT1* variants and schizophrenia but not bipolar disorder, and our results may be attributable to several methodological and population-specific factors. First, the relatively small sample size of bipolar disorder cases (1008 bipolar disorder patients and 2127 controls) in the Japanese cohort likely limited statistical power to detect modest effects of common variants. Second, the absence of age-stratified analyses may have masked age-dependent genetic effects, as our study demonstrated stronger associations in older individuals. Third, the Japanese study examined different tagging polymorphisms within *SIRT1*, which may capture distinct linkage disequilibrium patterns and functional variation compared with rs12415800. Finally, despite both cohorts being East Asian, genetic background and allele frequency distributions differ between Japanese and Taiwanese populations, potentially influencing variant–phenotype associations. Together, these considerations suggest that *SIRT1*-related genetic effects are sensitive to study design, SNP selection and ancestry, and highlight the need for adequately powered, stratified analyses in diverse populations.

From a broader perspective, these findings have important implications for future research in psychiatric genetics within a transdiagnostic framework. Incorporating rs12415800 into polygenic risk scores tailored for East Asian populations may improve risk stratification across mood disorder spectra rather than within a single diagnostic category. Given the multifunctional roles of the *SIRT1* enzyme in circadian regulation, metabolic signalling and cellular stress responses, future studies should prioritise intermediate phenotypes – such as sleep disturbance, metabolic dysregulation and stress reactivity – as potential mediators linking genetic variation to clinical outcomes. While earlier studies primarily reported associations between *SIRT1* and MDD or schizophrenia,^9,21^ our observation of a relationship between *SIRT1* and bipolar disorder supports the notion that *SIRT1* may confer shared vulnerability across multiple psychiatric conditions, consistent with emerging transdiagnostic models of mental disorders. Given that the rs12415800 risk A allele near *SIRT1* has been linked to reduced *SIRT1* mRNA expression and increased vulnerability to mood disorders,^18,32^ enhancing *SIRT1* and AMPK pathways may be particularly relevant. Resveratrol is a naturally occurring polyphenolic compound found in grapes, berries, peanuts and red wine, and has been widely investigated for its potential antioxidant, anti-inflammatory, mitochondrial and SIRT1/AMPK-modulating effects.^36^ These mechanisms are relevant to bipolar disorder, in which altered energy metabolism, circadian rhythm disruption, inflammatory activation and impaired stress resilience may contribute to mood instability. By enhancing *SIRT1* and AMPK pathways, resveratrol may improve cellular energy homeostasis, mitochondrial function, inflammatory regulation and circadian stability. Thus, modulation of the *SIRT1*–AMPK axis represents a biologically plausible adjunctive strategy for targeting metabolic, inflammatory and rhythm-related abnormalities in bipolar disorder.^12^

The use of NHIRD-based psychiatric diagnoses offers several advantages, including comprehensive population coverage, longitudinal follow-up and standardised reimbursement-driven documentation. The large sample size and ancestry-homogeneous cohort also provide substantial statistical power and reduce confounding by population structure. Importantly, mood disorder diagnoses in NHIRD reflect real-world clinical practice rather than research-driven assessments, enhancing translational relevance. To improve diagnostic specificity, we adopted conservative case definitions requiring repeated psychiatrist-recorded out-patient diagnoses or an in-patient diagnosis, consistent with prior NHIRD research.^27,28^ Several limitations merit consideration. First, although repeated-claim algorithms are commonly used in NHIRD studies, the specific algorithm used here has not been formally validated against structured diagnostic interviews for bipolar disorder, schizophrenia, or schizo-affective disorder in this linked cohort. Diagnostic misclassification and evolving diagnoses between bipolar disorder and schizophrenia-spectrum disorders therefore remain possible. The sensitivity analysis reduced this concern for the overall and age-stratified findings, but the sex-stratified association was not stable: the significant male AA association in the primary analysis was attenuated after exclusion, whereas a significant female AG association emerged. Accordingly, sex-specific results should be regarded as exploratory and interpreted cautiously. Second, mood disorder phenotypes were defined categorically and could not capture clinical heterogeneity such as episode recurrence, symptom severity, or treatment response. Third, environmental exposures – including psychosocial stress, sleep patterns, physical activity and early-life adversity – were not comprehensively measured and therefore could not be evaluated as moderators of genetic risk. These dimensions may be particularly relevant for *SIRT1*, given its involvement in stress adaptation and neuroplasticity. Fourth, there is potential heterogeneity within mood disorder diagnoses. Accumulating genomic evidence indicates that mood disorder subtypes exhibit partially distinct genetic architectures, characterised by shared polygenic liability alongside subtype-specific enrichment patterns, particularly differentiating bipolar I disorder, bipolar II disorder and MDD.^37^ Consequently, analyses that do not fully disentangle these subtypes – especially in claims-based data-sets where diagnostic misclassification may occur – may attenuate true genetic associations or obscure subtype-specific effects. Fifth, because TWB participants entered the cohort as adults, some individuals may have had MDD or bipolar disorder before TWB enrolment or before the available NHIRD observation window. Thus, the first recorded NHIRD diagnosis may not represent true illness onset. Accordingly, age-stratified findings should be interpreted as associations among participants enrolled at older versus younger ages, rather than as evidence for first-onset late-life MDD or bipolar disorder. Finally, as with all observational genetic association studies, functional studies are required to delineate precise biological mechanisms of the risk gene.

In conclusion, using a large TWB–NHIRD linked cohort, we found that rs12415800 near *SIRT1* was associated with bipolar disorder, particularly among older participants, although sex-specific findings were less consistent in the sensitivity analysis. No significant association with MDD was observed in the overall sample. These findings suggest that the effect of rs12415800 may differ across mood disorder phenotypes in Han Chinese populations.

## Supporting information

10.1192/bjo.2026.12114.sm001 Liu et al. supplementary material Liu et al. supplementary material

## Data Availability

The individual-level data used in this study are not publicly available and cannot be directly shared because of Taiwan Biobank (TWB) and National Health Insurance research Database (NHIRD) data-use regulations and privacy protections. Qualified researchers may apply for access through the TWB and relevant data custodians. To gain access, interested individuals should contact ‘biobank@gate.sinica.edu.tw.’ Analytic code, ICD code definitions and aggregate summary results are available from the corresponding author upon reasonable request and subject to institutional approval.

## Supplementary material

The supplementary material is available online at https://doi.org/10.1192/bjo.2026.12114

## Table 1 Long description

The table presents baseline characteristics of study participants according to rs12415800 genotype, with three genotype groups: GG, AG, and AA. It includes data on sex distribution, age, education level, smoking status, and alcohol consumption. The table has 7 rows and 5 columns. Column headers are rs12415800, GG, AG, AA, and p. Row labels include Male, Age, Education, Smoking, and Drinking. Each row provides specific values for each genotype group and a p-value indicating statistical significance. Notable trends include small but statistically significant differences in sex distribution and mean age across genotype groups, while education level, smoking status, and alcohol consumption show no significant differences.

Navigate back to Table 1.

## Table 2 Long description

The table presents the association between the rs12415800 genotype and major depressive disorder (MDD) across various demographic groups. It includes columns for Non-MDD and MDD with counts and percentages, odds ratios with 95% confidence intervals, and adjusted odds ratios (aOR) with 95% confidence intervals. The table is divided into rows for different genotypes (GG, AG, AA) and demographic categories (All, Age ≥50, Age <50, Male, Female). Each row provides specific data points for these categories. Notable trends include the comparison of genotype distributions between Non-MDD and MDD groups, with adjustments for age and sex where applicable.

Navigate back to Table 2.

## Table 3 Long description

The table presents data on the association between the SIRT1 rs12415800 polymorphism and bipolar disorder, categorized by overall sample, age groups, and sex. It includes columns for genotype (GG, AG, AA), non-bipolar disorder (n, %), bipolar disorder (n, %), odds ratio (95% CI), and adjusted odds ratio (aOR, 95% CI). The table has 12 rows and 5 columns. Row 1: All, GG, 46662 (98.90), 517 (1.10), Ref. Row 2: All, AG, 70775 (98.77), 881 (1.23), 1.12 (1.01-1.25), 1.12 (1.01-1.25). Row 3: All, AA, 26591 (98.69), 354 (1.31), 1.20 (1.05-1.38), 1.20 (1.05-1.37). Row 4: Age >=50, GG, 24286 (99.04), 236 (0.96), Ref. Row 5: Age >=50, AG, 36582 (98.82), 436 (1.18), 1.23 (1.05-1.44), 1.22 (1.04-1.44). Row 6: Age >=50, AA, 13683 (98.67), 184 (1.33), 1.38 (1.14-1.68), 1.38 (1.14-1.68). Row 7: Age <50, GG, 22376 (98.76), 281 (1.24), Ref. Row 8: Age <50, AG, 34193 (98.72), 445 (1.28), 1.04 (0.89-1.20), 1.04 (0.89-1.20). Row 9: Age <50, AA, 12908 (98.70), 170 (1.30), 1.05 (0.87-1.27), 1.05 (0.86-1.27). Row 10: Male, GG, 17148 (99.05), 165 (0.95), Ref. Row 11: Male, AG, 25507 (98.91), 280 (1.09), 1.14 (0.94-1.38), 1.14 (0.94-1.38). Row 12: Male, AA, 9510 (98.68), 127 (1.32), 1.34 (1.10-1.75), 1.39 (1.10-1.75). Row 13: Female, GG, 29514 (98.82), 352 (1.18), Ref. Row 14: Female, AG, 45268 (98.69), 601 (1.31), 1.11 (0.98-1.27), 1.11 (0.97-1.27). Row 15: Female, AA, 17081 (98.69), 227 (1.31), 1.11 (0.94-1.32), 1.11 (0.94-1.32).

Navigate back to Table 3.
