# Supplementary material for: Association of the major depressive disorder-associated locus rs12415800 with bipolar disorder in a Taiwan Biobank–NHIRD linked cohort
Source: BJPsych Open. 2026 Oct 10;12(6):e254. doi: 10.1192/bjo.2026.12114 (PMC13748679; doi:10.1192/bjo.2026.12114)
Supplement: Supplementary file 1 [file S2056472426121140sup001.docx]

**SUPPLEMENTARY**

**Supplementary Table S1** Age at first recorded NHIRD diagnosis across rs12415800 genotypes

| Age at first recorded NHIRD diagnosis (years), means$\pm$SD | GG | AG | AA | *p* |
| --- | --- | --- | --- | --- |
| MDD (n=15,481) | n=4,990 | n=7,557 | n=2,934 |  |
|  | 42.79$\pm$12.52 | 42.81$\pm$12.49 | 42.67$\pm$12.72 | 0.86 |
| BD (n=1,752) | n=517 | n=881 | n=354 |  |
|  | 40.42$\pm$12.85 | 41.00$\pm$12.62 | 41.83$\pm$13.21 | 0.28 |

Data are presented as mean$\pm$standard deviation (SD).

BD, bipolar disorder; MDD, major depressive disorder; NHIRD, National Health Insurance Research Database.

*P* values were calculated using ANOVA to compare differences across the three genotype groups.

**Supplementary Table S2** Baseline characteristics of the study participants according to rs12415800 genotypes, excluding individuals with schizophrenia or schizoaffective disorder

| rs12415800 | GG  (n=46,907) | AG  (n=71,215) | AA  (n=26,754) | *p* |
| --- | --- | --- | --- | --- |
| Male (n, %) | 17,196 (36.66) | 25,599 (35.95) | 9,560 (35.73) | **0.01** |
| Age, mean, SD | 49.55$\pm$11.42 | 49.43$\pm$11.35 | 49.33$\pm$11.41 | **0.03** |
| Education (n, %) |  |  |  | 0.10 |
| No/Non-formal | 92 (0.20) | 160 (0.22) | 43 (0.16) |  |
| Elementary school | 2,240 (4.78) | 3,255 (4.57) | 1,260 (4.71) |  |
| Junior high school | 3,477 (7.41) | 4,956 (6.96) | 1,851 (6.92) |  |
| Senior high/ vocational school | 13,306 (28.37) | 20,329 (28.55) | 7,634 (28.53) |  |
| College/University | 22,536 (48.04) | 34,431 (48.35) | 12,905 (48.24) |  |
| Graduate school | 5,256 (11.21) | 8,084 (11.35) | 3,061 (11.44) |  |
| Smoking (n, %) | 12,945 (27.60) | 19,339 (27.16) | 7,215 (26.97) | 0.12 |
| Drinking (n, %) | 2,845 (6.07) | 4,343 (6.10) | 1,631 (6.10) | 0.47 |

Statistical significance is indicated with bold type.

**Supplementary Table S3** Association between rs12415800 and risk of major depressive disorder (MDD), excluding individual with schizophrenia or schizoaffective disorder

|  | rs12415800 | Non-MDD (n, %) | MDD (n, %) | OR (95% CI) | aOR (95% CI) |
| --- | --- | --- | --- | --- | --- |
| **All** |  |  |  |  |  |
|  | GG | 42,087 (89.72) | 4,820 (10.28) | Ref. | Ref. (Adjusted Sex and Age) |
|  | AG | 63,925 (89.76) | 7,290 (10.24) | 1.00 (0.96－1.03) | 0.99 (0.96－1.03) |
|  | AA | 23,941 (89.49) | 2,813 (10.51) | 1.03 (0.98－1.08) | 1.02 (0.98－1.08) |
| **Age >=50** |  |  |  |  |  |
|  | GG | 21,696 (88.75) | 2,750 (11.25) | Ref. | Ref. (Adjusted Sex) |
|  | AG | 32,653 (88.58) | 4,209 (11.42) | 1.02 (0.97－1.07) | 1.01 (0.96－1.07) |
|  | AA | 12,155 (88.13) | 1,637 (11.87) | **1.06 (1.00－1.13)** | **1.06 (1.00－1.13)** |
| **Age < 50** |  |  |  |  |  |
|  | GG | 20,391 (90.78) | 2,070 (9.22) | Ref. | Ref. (Adjusted Sex) |
|  | AG | 31,272 (91.03) | 3,081 (8.97) | 0.97 (0.92－1.03) | 0.97 (0.91－1.03) |
|  | AA | 11,786 (90.93) | 1,176 (9.07) | 0.98 (0.91－1.06) | 0.98 (0.91－1.06) |
| **Male** |  |  |  |  |  |
|  | GG | 15,930 (92.64) | 1,266 (7.36) | Ref. | Ref. (Adjusted Age) |
|  | AG | 23,658 (92.42) | 1,941 (7.58) | 1.03 (0.96－1.11) | 1.03 (0.96－1.11) |
|  | AA | 8,882 (92.91) | 678 (7.09) | 0.96 (0.87－1.06) | 0.96 (0.87－1.06) |
| **Female** |  |  |  |  |  |
|  | GG | 26,157 (88.04) | 3,554 (11.96) | Ref. | Ref. (Adjusted Age) |
|  | AG | 40,267 (88.27) | 5,349 (11.73) | 0.98 (0.93－1.02) | 0.98 (0.94－1.02) |
|  | AA | 15,059 (87.58) | 2,135 (12.42) | 1.04 (0.99－1.10) | 1.05 (0.99－1.11) |

Ref: Reference group; OR: odds ratio; CI: confidence interval; aOR: Adjusted odds ratio

Statistical significance is indicated with bold type.

**Supplementary Table S4** Association between rs12415800 and risk of bipolar disorder (BD), excluding individuals with schizophrenia or schizoaffective disorder

|  | rs12415800 | Non-BD (n, %) | BD (n, %) | OR (95% CI) | aOR (95% CI) |
| --- | --- | --- | --- | --- | --- |
| **All** |  |  |  |  |  |
|  | GG | 46,478 (99.09) | 429 (0.91) | Ref. | Ref. (Adjusted Sex and Age) |
|  | AG | 70,461 (98.94) | 754 (1.06) | **1.16 (1.03－1.31)** | **1.16 (1.03－1.30)** |
|  | AA | 26,468 (98.93) | 286 (1.07) | **1.17 (1.01－1.36)** | **1.17 (1.01－1.36)** |
| **Age >=50** |  |  |  |  |  |
|  | GG | 24,236 (99.14) | 210 (0.86) | Ref. | Ref. (Adjusted Sex) |
|  | AG | 36,472 (98.94) | 390 (1.06) | **1.23 (1.04－1.46)** | **1.23 (1.04－1.46)** |
|  | AA | 13,632 (98.84) | 160 (1.16) | **1.35 (1.10－1.67)** | **1.35 (1.10－1.66)** |
| **Age < 50** |  |  |  |  |  |
|  | GG | 22,242 (99.02) | 219 (0.98) | Ref. | Ref. (Adjusted Sex) |
|  | AG | 33,989 (98.94) | 364 (1.06) | 1.09 (0.92－1.29) | 1.09 (0.92－1.29) |
|  | AA | 12,836 (99.03) | 126 (0.97) | 1.00 (0.80－1.24) | 0.99 (0.80－1.24) |
| **Male** |  |  |  |  | Adjusted Age |
|  | GG | 17,059 (99.20) | 137 (0.80) | Ref. | Ref. |
|  | AG | 25,374 (99.12) | 225 (0.88) | 1.10 (0.89－1.37) | 1.10 (0.89－1.37) |
|  | AA | 9,462 (98.97) | 98 (1.03) | 1.29 (0.99－1.67) | 1.29 (0.99－1.67) |
| **Female** |  |  |  |  | Adjusted Age |
|  | GG | 29,419 (99.02) | 292 (0.98) | Ref. | Ref. |
|  | AG | 45,087 (98.84) | 529 (1.16) | **1.18 (1.02－1.36)** | **1.18 (1.02－1.36)** |
|  | AA | 17,006 (98.91) | 188 (1.09) | 1.11 (0.93－1.34) | 1.11 (0.93－1.34) |

Ref: Reference group; CI: confidence interval; aOR: Adjusted odds ratio

Statistical significance is indicated with bold type.
